# Consolidation and seepage solutions in enclosure space based on non-zero-constant values boundary

**DOI:** 10.1371/journal.pone.0301581

**Published:** 2024-05-20

**Authors:** Liangjin Chen, Zi-kun Gao, Feixin Chen

**Affiliations:** 1 Meizhouwan Vocational Technology College, Putian, China; 2 Department of Civil Engineering of Lishui University, Lishui, China; 3 Southwest Zhejiang Institute of Prevent and Control of Geological Disasters, Lishui, China; 4 Guangdong Polytechnic Normal University, Guangzhou, China; Tribhuvan University, NEPAL

## Abstract

Research is ongoing to find solutions to the problem of Consolidation and seepage in saturated clay in enclosure space. Firstly, the boundary of non-zero-constant values is established, considering the seepage boundary of the clay is affected by pumping water or lowering boundary pressure on the site. Secondly, the differential equation is established to reflect the spatial and temporal variations of excess pore water pressure dissipation in the clay in enclosure space, and the solution is derived using variable separation methods. Finally, based on results of the solution derived, contour maps of the water pressure are drawn corresponding with the different inhomogeneous boundary conditions.

## 1 Introduction

Methods of foundation treatment such as vacuum combined with surcharge preloading and soil-squeezing pile can produce excess pore water pressure (EPWP) in saturated clay, and the dissipation of the pressure affects the bearing capacity of the foundation soil. It also affects the rate of consolidation compression.

The EPWP and its dissipation are explored for underground construction in saturated clay based on the mechanism of piling compaction [1]. Research results [2] also suggest that the bearing capacity of piles increases with the advance of the consolidation, and the average degree of consolidation is linearly related to the bearing capacity of the pile foundation. In the article [3], EPWP caused by installing piles in soft clay is studied by introducing logarithmic strain parameters and considering large deformation and softening characteristics.

Articles [4] analyzes the distribution and size of excess pore water pressure in soil, establishes the model of soil reconsolidation between piles, and compiles the calculation program with the three-dimensional consolidation and variation theory. The calculated growth rate of consolidation between piles is compared with that of pile bearing capacity measured. The results of calculation are in good agreement with that of soil consolidation rate between piles. The relationship between consolidation degree and bearing capacity, to some extent, can be considered that the change of consolidation degree and bearing capacity with time is corresponding, so this paper studies the orthotropic (transverse isotropic) soil consolidation solution between pile groups in a closed environment from the perspective of soil consolidation. Articles [5–7]research the lateral load influence on behaviour of negative skin friction on circular and square piles, hybrid energy piles as a smart and sustainable foundation and numerical analysis of seepage failure modes of sandy soils within a cylindrical cofferdam.

In articles [8–10], solutions of EPWP at the initial moment due to pile compaction and its dissipation have been derived and analyzed based on homogeneous boundary hypothesis. In this paper, the definite condition is established to find the consolidation and seepage solutions in enclosure space based on non-zero-constant values boundary.

The literature [11] applies the finite difference method to numerically simulate groundwater flow to analyze the seepage failure mode of non-cohesive sand inside a cofferdam. The study investigates the influence of cofferdam radius, internal soil friction, and soil expansiveness on the failure mode. The seepage flow and seepage failure modes obtained by numerical methods are presented under different working conditions. The literature [12] proposes a solution using a hybrid pile type, investigates the mechanical behavior of hybrid piles with strain gauges installed along the bearing during load tests, and presents a numerical simulation method for energy hybrid pile behavior. The literature [13] applies the finite difference method to numerically simulate groundwater flow to analyze the seepage failure mode of non-cohesive sand inside a cofferdam. The study investigates the influence of cofferdam radius, internal soil friction, and soil expansiveness on the failure mode. The seepage flow and seepage failure modes obtained by numerical methods are presented under different working conditions.

Drawing on the research methods of the above literature, this paper will establish a finite element model for the corresponding engineering example, use Matlab code programming to draw contour maps of pore water pressure dissipation, and compare and verify the correctness and convergence of the theoretical solutions derived in this paper.

## 2 Mechanical model and solution

**Fig 1:**
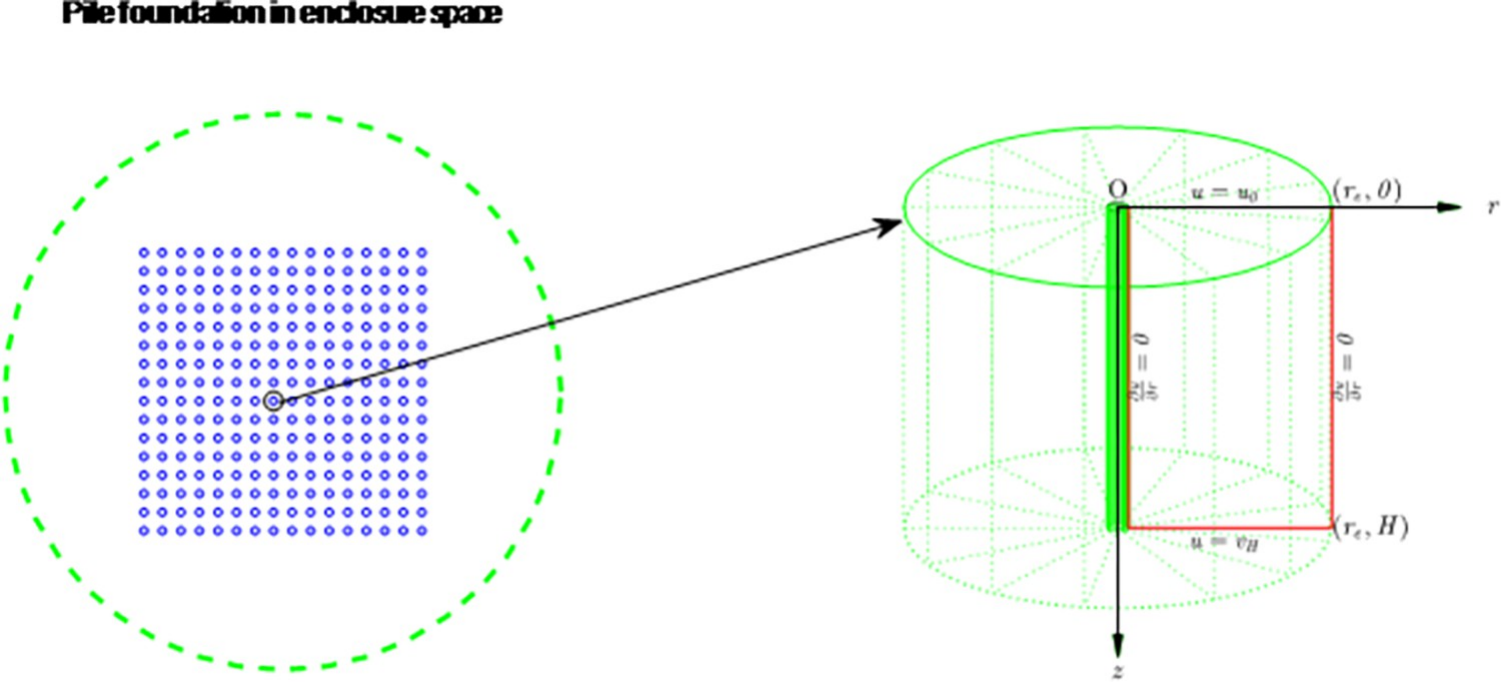
Consolidation soil model from enclosure space.

The boundary and partial differential equation [14] are given as Eqs (1–3) and shown in the consolidation soil model from enclosure space Fig 1. The so-called closed environment refers to the pile group foundation distributed in a large area, and the site around the pile foundation is not well-drained or the soil around the site is other built civil engineering foundation with low permeability after foundation treatment. In the closed environment, the consolidated seepage flow in the pile group site has spatial characteristics, but the seepage flow in the area outside the project site is basically not affected by the pile compression of the project under construction. Therefore, the consolidation characteristics of soil between piles are different from the soil consolidation around a single pile and the soil consolidation between piles in the pile group foundation under general site conditions, and need to be studied separately according to its unique boundary conditions.

$$\frac{\partial u}{\partial t}=C_{h}\frac{1}{r}\frac{\partial }{\partial r}(r\frac{\partial u}{\partial r})+C_{v}\frac{\partial^{2}u}{\partial z^{2}}$$ (1)

$${u|}_{t=0}=f(r,z)$$ (2)

$${u|}_{z=0}=u_{0},{u|}_{z=H}=u_{H},{\frac{\partial u}{\partial r}|}_{r=r_{w}}=0,{\frac{\partial u}{\partial r}|}_{r=r_{e}}=0$$ (3)

where *u* is the function of excess pore water pressure, *C*_h_ and *C*_v_ are radial and vertical direction consolidation coefficients respectively, *r* and *z* are horizontal and vertical coordinates respectively, *f*(*r*,*z*) is the initial condition function of the pressure, *r*_*w*_ is the well radius, *r*_*e*_ is the influence radius of *u*|_*t* = 0_ = *f*(*r*,*z*), *H* is the thickness of consolidated soil, *u*_0_ is the water pressure value at *z* = 0, and *u*_*H*_ is the water pressure value at *z* = *H*.

To homogenize the boundary conditions, *u*(*r*,*z*,*t*) can be expressed as Eq (4)

u=V+W (4)

**Fig 2:**
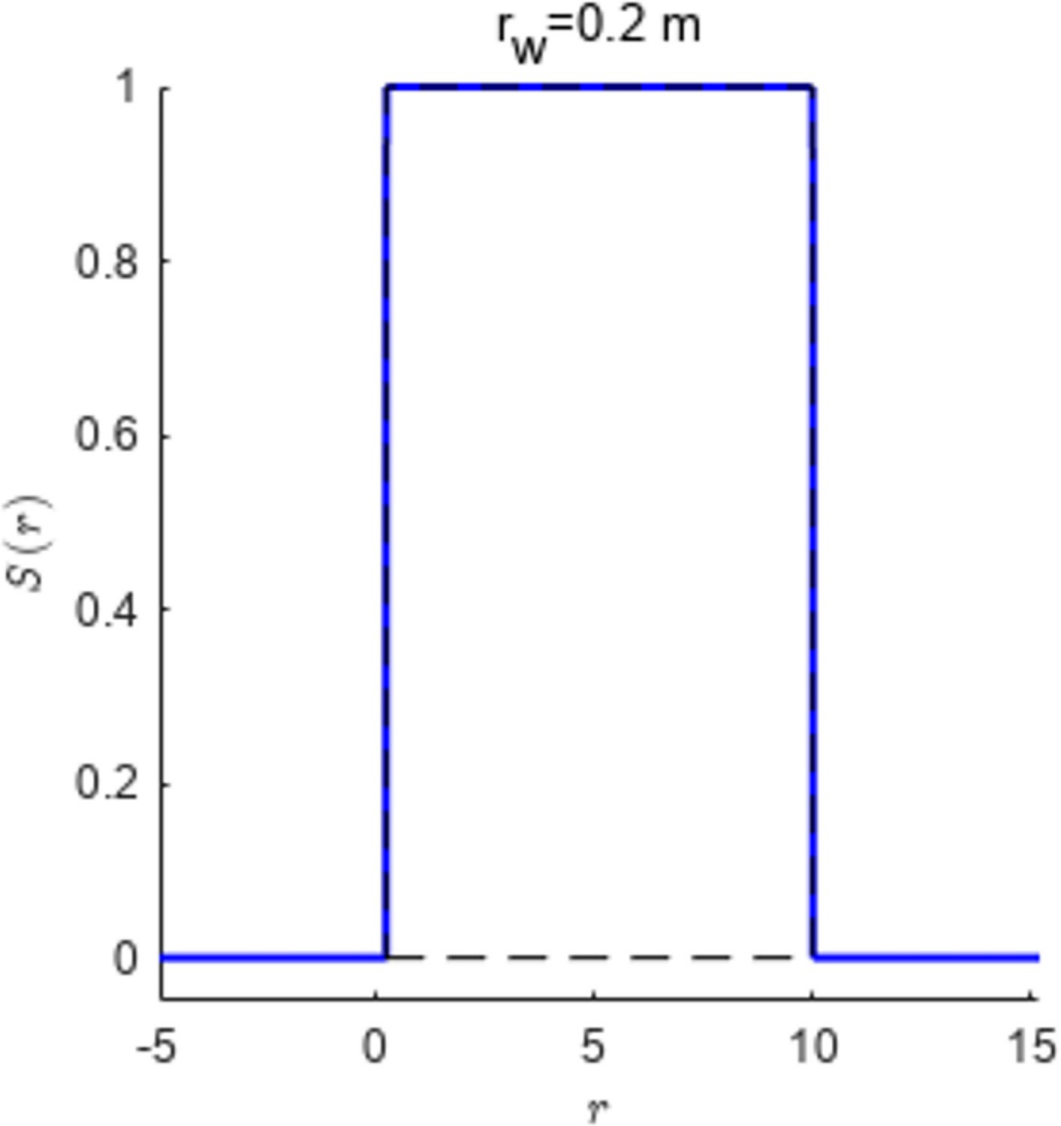
Step function.

Where *u* = *u*(*r*,*z*,*t*), *V* = *V*(*r*,*z*,*t*), $W=W(r,z)=S_{sfr}(r)\cdot (z\times \frac{u_{H}-u_{0}}{H}+u_{0})$, *S*_*sfr*_ is the function expressed as Eq (5) and in Fig 2 when the well radius is *r*_*w*_.


$$S_{sfr}(r)=\{\begin{array}{l} 1,\;r_{w}<r<r_{e}\\ 0,\;r\geq r_{e}\;or\;r\leq r_{w} \end{array}$$ (5)

Substituting Eq (4) into equation Eq (1) can obtain Eq (6)

$$\frac{\partial V}{\partial t}=C_{h}\frac{1}{r}\frac{\partial }{\partial r}(r\frac{\partial V}{\partial r})+C_{v}\frac{\partial^{2}V}{\partial z^{2}}-\frac{\partial W(r,z)}{\partial t}$$ (6)

where ${\partial V/\partial r|}_{r=r_{w}}=0,{\partial V/\partial r|}_{r=r_{e}}=0$, *V*|_z = 0_ =0,*V*|_*z* =*H*_ = 0, and the initial condition *V*(*r*,*z*,0) = *f*(*r*,*z*)−*W*.

Let *V*(*r*,*z*,*t*) = *R*(*r*)*Z*(*z*)*T*(*t*), and Eq (6) can be expressed as Eq (7)

$$RZT^{\prime }-C_{h}DZT-C_{v}TRZ^{\prime \prime }=0$$ (7)

where $D=R^{\prime \prime }+\frac{R^{\prime }}{r}$, $T^{\prime }=\frac{dT}{dt},$ $R^{\prime \prime }=\frac{dR^{\prime }}{dr}$, $Z^{\prime \prime }=\frac{d^{2}Z}{dz^{2}}$.

By introducing parameter *μ*, Eq (7) can be expressed as Eq (8).

$$\frac{Z^{\prime \prime }}{Z}=\frac{RT^{\prime }-C_{h}DT}{C_{v}TR}=-\mu \Rightarrow \{\begin{array}{l} Z^{\prime \prime }+\mu Z=0\\ RT^{\prime }-C_{h}DT+\mu C_{v}TR=0 \end{array}$$ (8)

For *RT*′−*C*_*h*_*DT*+*μC*_*v*_*TR* = 0 of Eq (8), parameter *λ* can be introduced to obtain Eq (9).

$$\frac{T^{\prime }}{C_{h}T}=\frac{D-\mu \frac{C_{v}}{C_{h}}R}{R}=-\lambda \Rightarrow \{\begin{array}{l} T^{\prime }+\lambda C_{h}T=0\\ R^{\prime \prime }+\frac{R^{\prime }}{r}+(\lambda -\mu \frac{C_{v}}{C_{h}})R=0\Rightarrow R^{\prime \prime }+\frac{R^{\prime }}{r}+\alpha^{2}R=0 \end{array}$$ (9)

Eqs (8 and 9) satisfy the boundary condition and the initial condition of Eq (6).

The differential equations shown in Eqs (8 and 9) can be solved to obtain Eqs (10–12)

$$Z_{k}(z)=C_{k}\sin\sqrt{\mu_{k}}z$$ (10)

$$\left\{ \begin{array}{l} R_{i}(r)=C_{i}J_{0}(\alpha_{i}r)-C_{i}\frac{J_{1}(\alpha_{i}r_{e})}{Y_{1}(\alpha_{i}r_{e})}Y_{0}(\alpha_{i}r)\;\left(\alpha_{i}\neq 0\right)\\ R_{0}(r)=Const.\;\left(\alpha_{i}=0\right) \end{array}\right.$$ (11)

$$T(t)=Ae^{-\lambda C_{h}t}$$ (12)

where $\mu_{k}=\frac{\pi^{2}}{4}\frac{{\left(2k-1\right)}^{2}}{H^{2}}\;\left(k=1,2,3,\cdots \right)$, *Y*_0_、*Y*_1_、 *J*_0_ and *J*_1_ are Bessel function. *α*_*i*_(*i* = 1,2,3,⋯) are positive eigenvalues of $J_{1}(\alpha r_{0})Y_{1}(\alpha r_{e})-J_{1}(\alpha r_{e})Y_{1}(\alpha r_{0})=0$. The eigenfunction $\left\{\sin(\sqrt{\mu_{k}}z)\;,[J_{0}(\alpha_{i}r)-\frac{J_{1}(\alpha_{i}r_{e})}{Y_{1}(\alpha_{i}r_{e})}Y_{0}(\alpha_{i}r)]\sin(\sqrt{\mu_{k}}z)\right\}$ (*k* = 1,2,3⋯,*i* = 1,2,3⋯) can be obtained based on Eqs (10–12). We can verify that $\left\{\sin(\sqrt{\mu_{k}}z)\;,[J_{0}(\alpha_{i}r)-\frac{J_{1}(\alpha_{i}r_{e})}{Y_{1}(\alpha_{i}r_{e})}Y_{0}(\alpha_{i}r)]\sin(\sqrt{\mu_{k}}z)\right\}$ (*k* = 1,2,3⋯,*i* =1,2,3⋯) forms a completely orthogonal sequence.

Combining the initial conditions for the consolidation seepage and complete orthogonality described above, series solution of *V*(*r*,*z*,*t*) can be derived and shown as Eq (13)

$$\begin{array}{l} V(r,z,t)=\sum_{k=1}^{\infty }C_{k,0}\sin(\sqrt{\mu_{k}}z)e^{-\lambda_{k,0}C_{h}t}\\ \;+\sum_{i=1}^{\infty }\sum_{k=1}^{\infty }C_{k,i}M_{i}\sin(\sqrt{\mu_{k}}z)e^{-\lambda_{k,i}C_{h}t} \end{array}$$ (13)

where $M_{i}=[J_{0}(\alpha_{i}r)-\frac{J_{1}(\alpha_{i}r_{e})}{Y_{1}(\alpha_{i}r_{e})}Y_{0}(\alpha_{i}r)]$, *λ*_*K*,0_ = *nμ*_*k*_, $\lambda_{k,i}={\alpha_{i}}^{2}+n\mu_{k}$; *k*, *i* = 1,2,3,⋯.

$$C_{k,i}=\frac{\int_{r_{0}}^{r_{e}}\int_{0}^{H}V(r,z,0)M_{i}\sin(\sqrt{\mu_{k}}z)rdrdz}{\int_{r_{w}}^{r_{e}}\frac{H}{2}\frac{{\left[J_{0}(\alpha_{i}r)Y_{1}(\alpha_{i}r_{e})-Y_{0}(\alpha_{i}r)J_{1}(\alpha_{i}r_{e})\right)}^{2}}{{J_{1}}^{2}(\alpha_{i}r_{e})}rdr},C_{k,0}=\frac{\int_{r_{0}}^{r_{e}}\int_{0}^{H}V(r,z,0)r\sin(\sqrt{\mu_{k}}z)drdz}{\int_{r_{0}}^{r_{e}}\int_{0}^{H}r\sin^{2}(\sqrt{\mu_{k}}z)drdz},$$

According to Eqs (4) and (13), the theoretical solution of partial differential Eq (1) can be expressed as Eq (14).

$$u=V+S_{sfr}(r)\cdot (z\times \frac{u_{H}-u_{0}}{H}+u_{0})$$ (14)

The average consolidation degree can be defined as Eq (15)

$$U(t)=1-\int_{0}^{r_{e}}\int_{0}^{H}urdrdz/\int_{0}^{r_{e}}\int_{0}^{H}frdrdz$$ (15)

## 3 Calculation and analysis of engineering example

### 3.1 Physical and geometric parameters

According to a test pile foundation [15], geometrical parameters are *H* = 24.5m, *r*_*w*_ = 0.2m and *r*_*e*_ = 10m.

Based on parameters shown in Table 1 and taking soil layer thickness as the weight, the mean values of calculation arguments are taken as follows

$$\gamma^{\prime }=\frac{18\times 7+17.2\times 10+18.3\times 7.5}{24.5}-9.8=7.97\mathrm{kN};$$

$$E_{s}=\frac{3000\times 7+2800\times 10+5000\times 7.5}{24.5}=3.53\times 10^{3}\mathrm{kN};$$

$$k_{v}=\frac{0.015\times 7+0.01\times 10+0.015\times 7.5}{24.5}\times 0.01=1.30\times 10^{-4}m•d^{-1}$$

$$k_{h}=\frac{0.15\times 7+0.1\times 10+0.15\times 7.5}{24.5}\times 0.01=12.96\times 10^{-4}m•d^{-1}$$

$$C_{v}=\frac{(1+\nu )k_{v}E_{s}}{3(1-\nu )\gamma_{w}}=0.0309m^{2}d^{‐1};C_{h}=\frac{(1+\nu ){k_{h}}^{\prime }E_{s}}{3(1-\nu )\gamma_{w}}=0.206m^{2}d^{‐1}.$$

where *γ*_*w*_ is the bulk density of water, and *v* = 0.48 is Poisson’s ratio of the saturated clay.

**Table 1:** Physical and mechanical parameters of soil.

Soil layer	Thick/ m	*γ*/kN m^−3^	*e*	*K*_h_/ cm·d ^-1^	*K*_v_/ cm·d ^-1^	*E*_*s*_/MPa
Silty clay	7	18.0	1.15	0.15	0.015	3.0
Clay	10	17.2	1.38	0.10	0.010	2.8
Loam	14	18.3	1.02	0.15	0.015	5.0

According to the article [8], the initial condition of Eq (2) has been expressed as Eq (16)

$$f(r,z)=\frac{r_{e}-r}{r_{e}-r_{w}}(\gamma^{\prime }z+c_{u})\;(r_{w}\leq r\leq r_{e})$$ (16)

where *c*_*u*_ = 12.4kPa.

**Fig 3:**
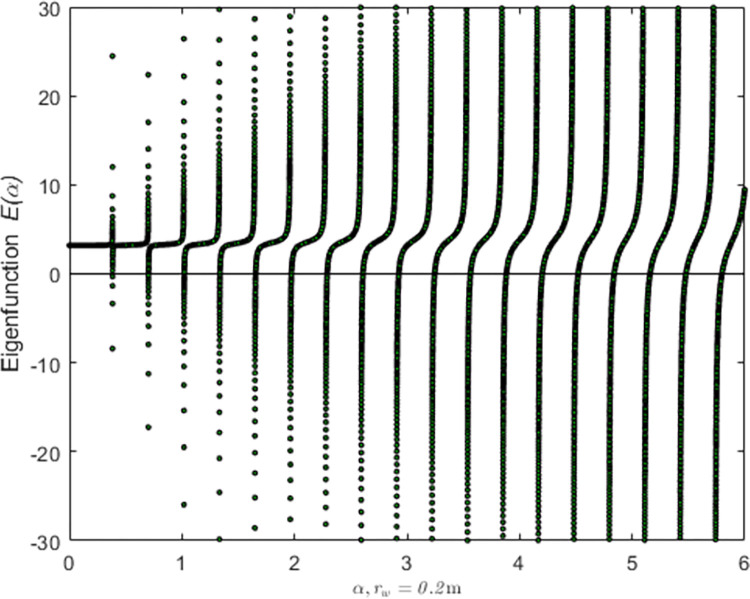
Eigenvalues when the well radius is 0.2m.

According to the characteristic equation $J_{1}(\alpha r_{0})Y_{1}(\alpha r_{e})-J_{1}(\alpha r_{e})Y_{1}(\alpha r_{0})=0$, positive eigenvalues can be obtained with iteration method as shown in Fig 3.

### 3.2 Theoretical calculation and analysis

**Fig 4:**
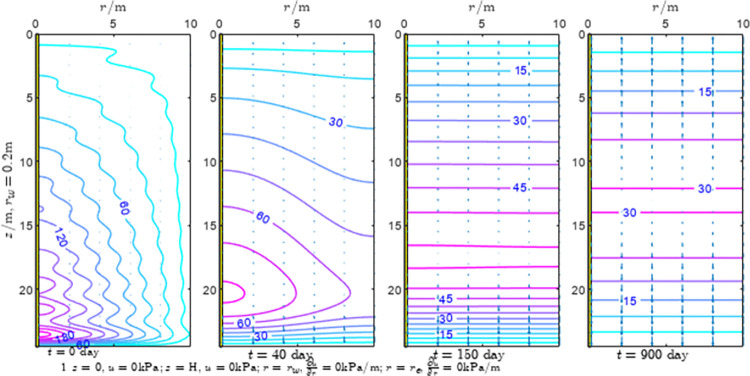
Spatial and temporal variation of consolidation seepage when the well radius is 0.2m.

**Fig 5:**
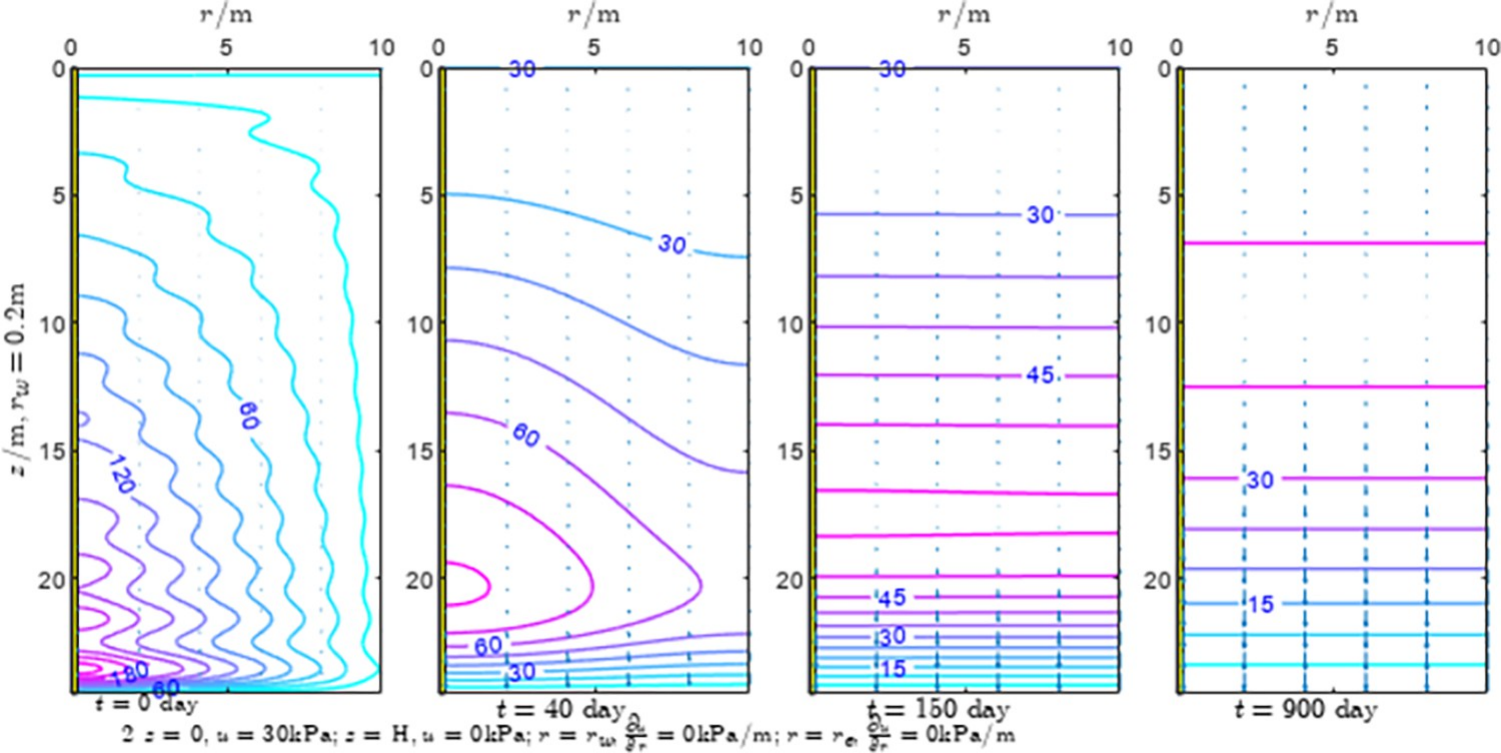
Spatial and temporal variation of consolidation seepage when the well radius is 0.2m.

**Fig 6:**
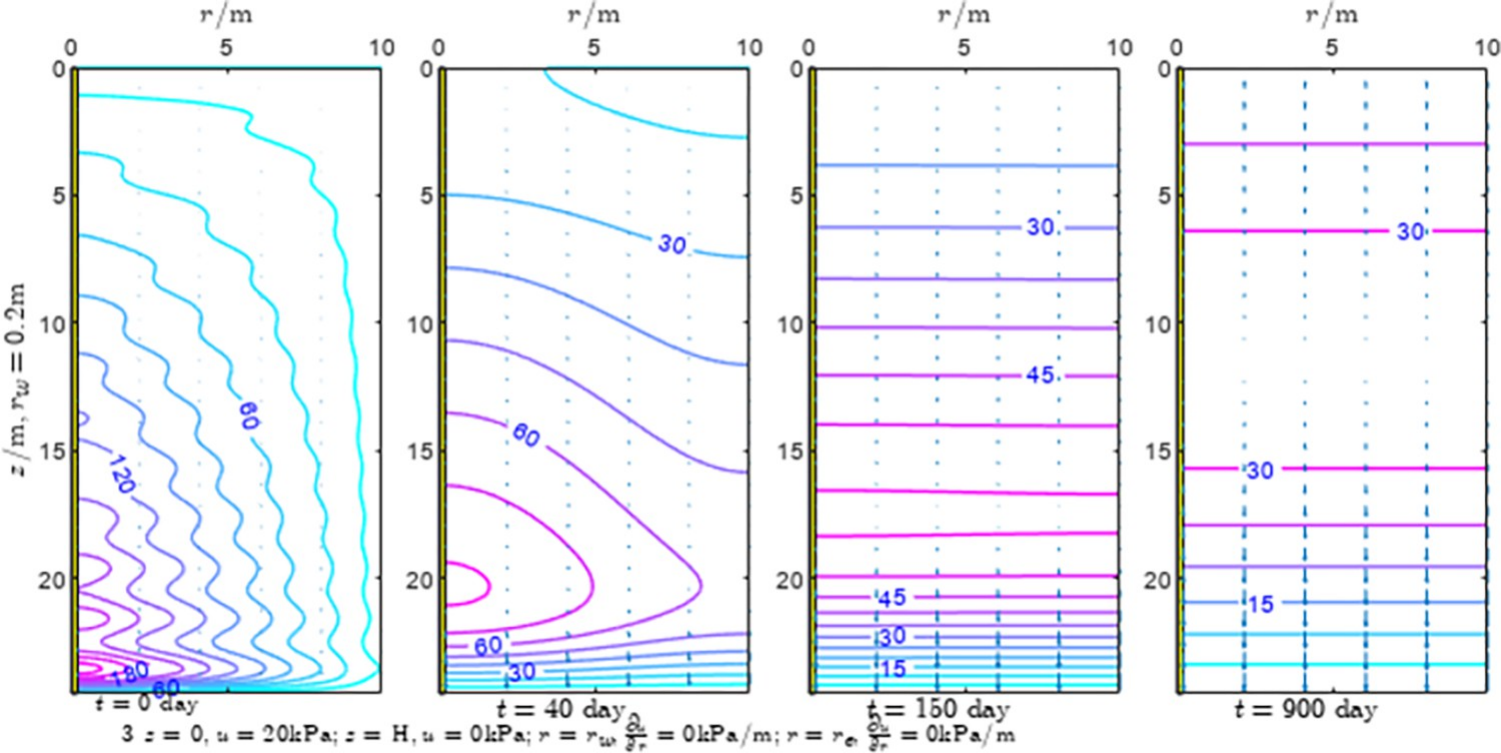
Spatial and temporal variation of consolidation seepage when the well radius is 0.2m.

**Fig 7:**
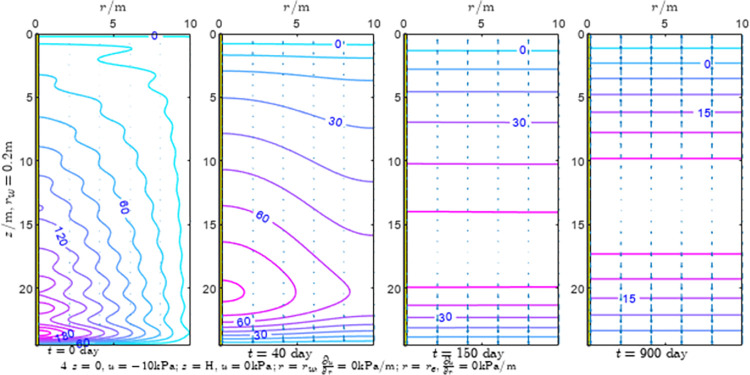
Spatial and temporal variation of consolidation seepage the well radius is 0.2m.

**Fig 8:**
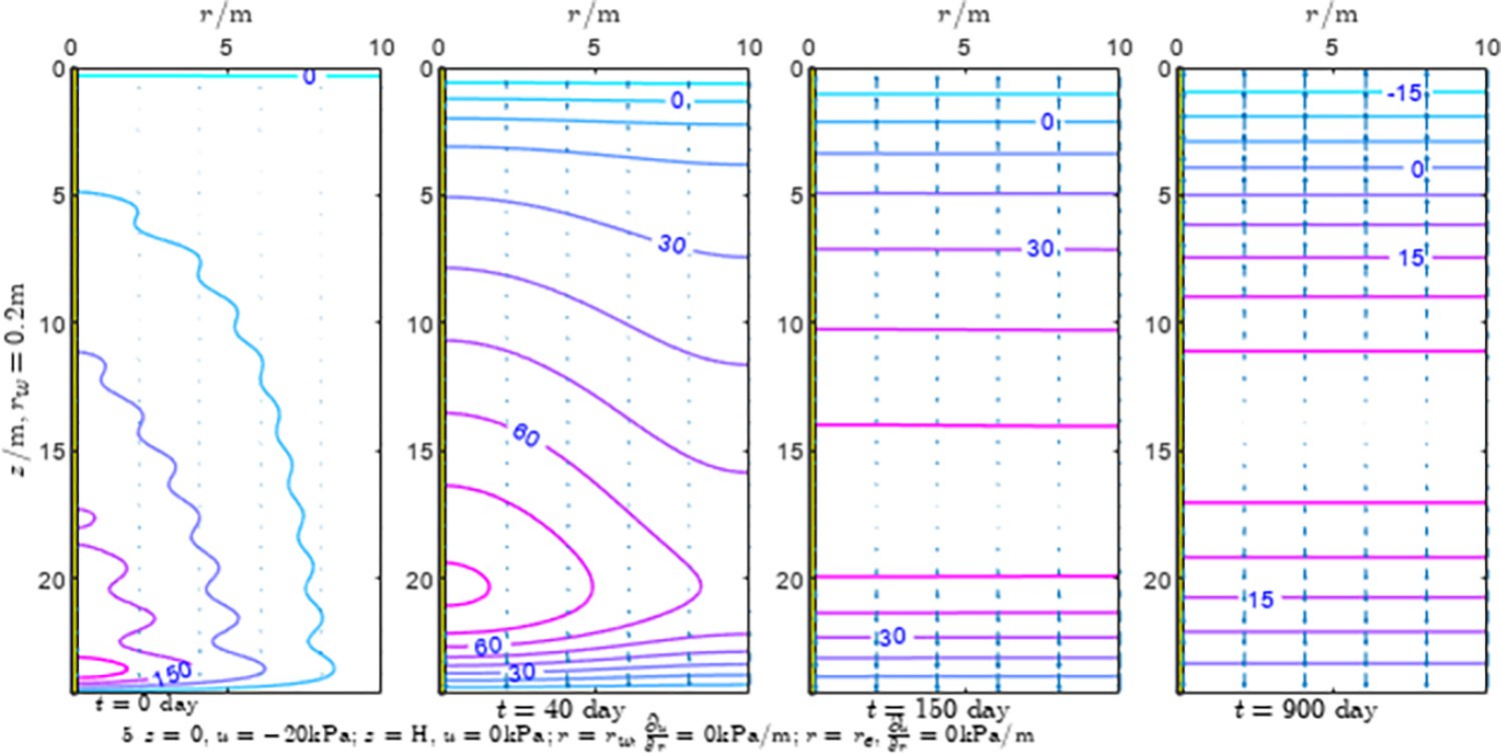
Spatial and temporal variation of consolidation seepage when the well radius is 0.2m.

**Fig 9:**
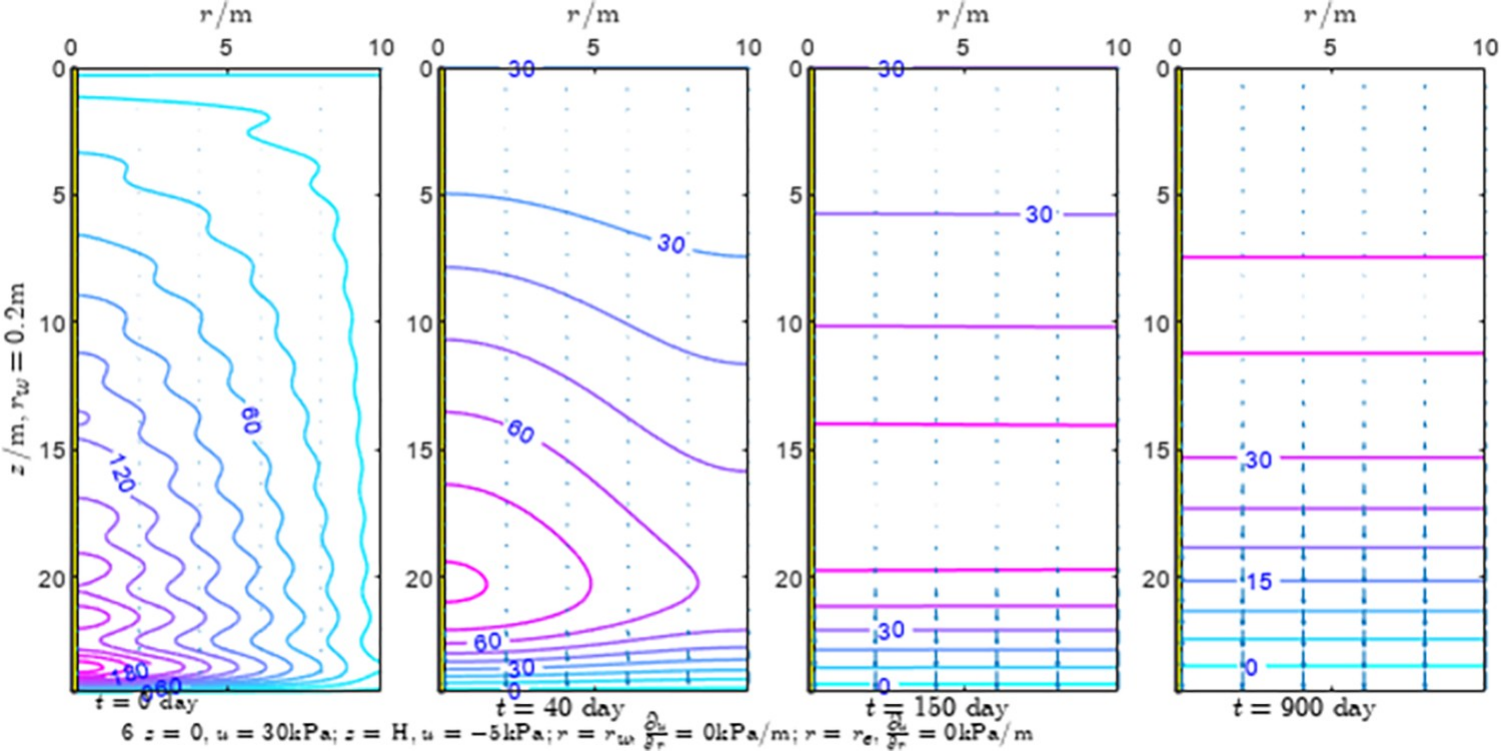
Spatial and temporal variation of consolidation seepage when the well radius is 0.2m.

**Fig 10:**
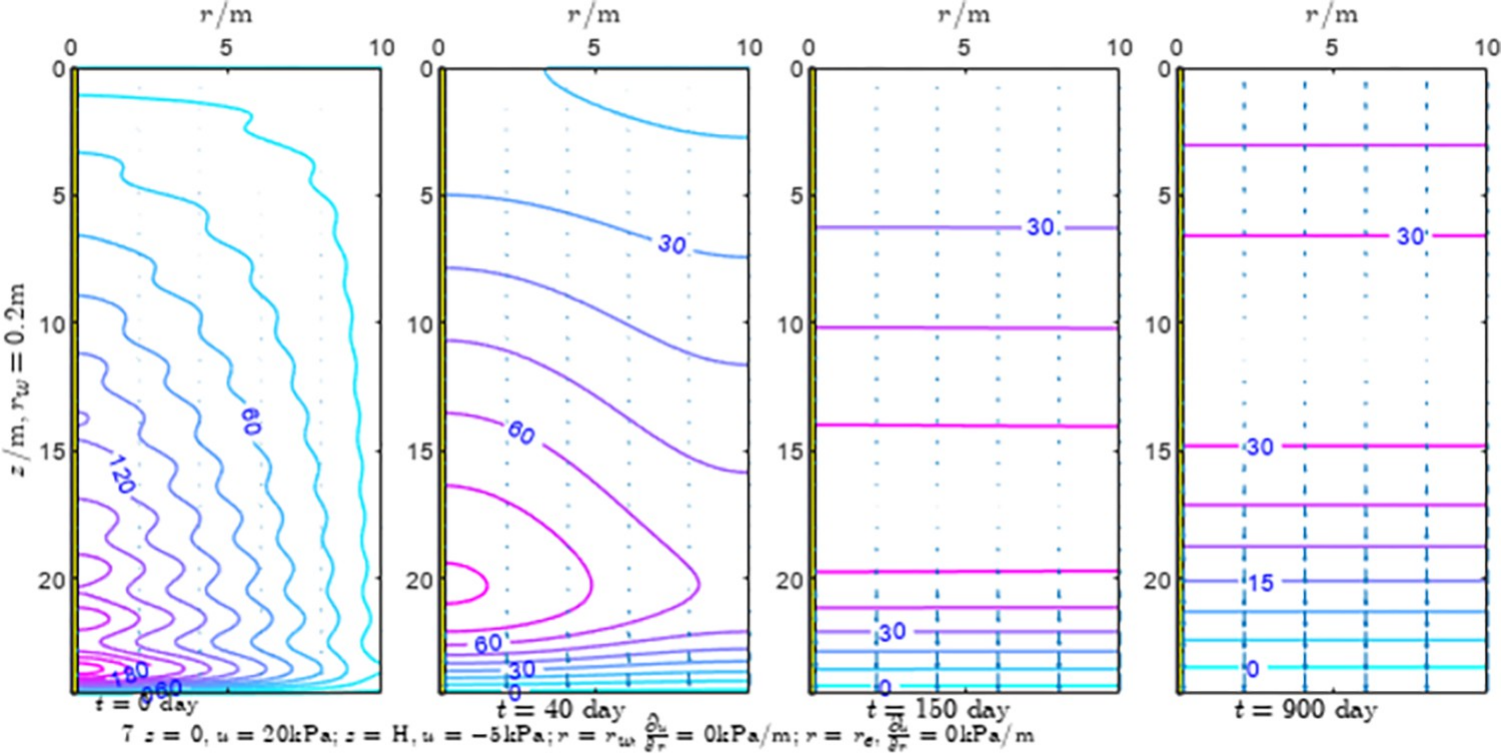
Spatial and temporal variation of consolidation seepage when the well radius is 0.2m.

**Fig 11:**
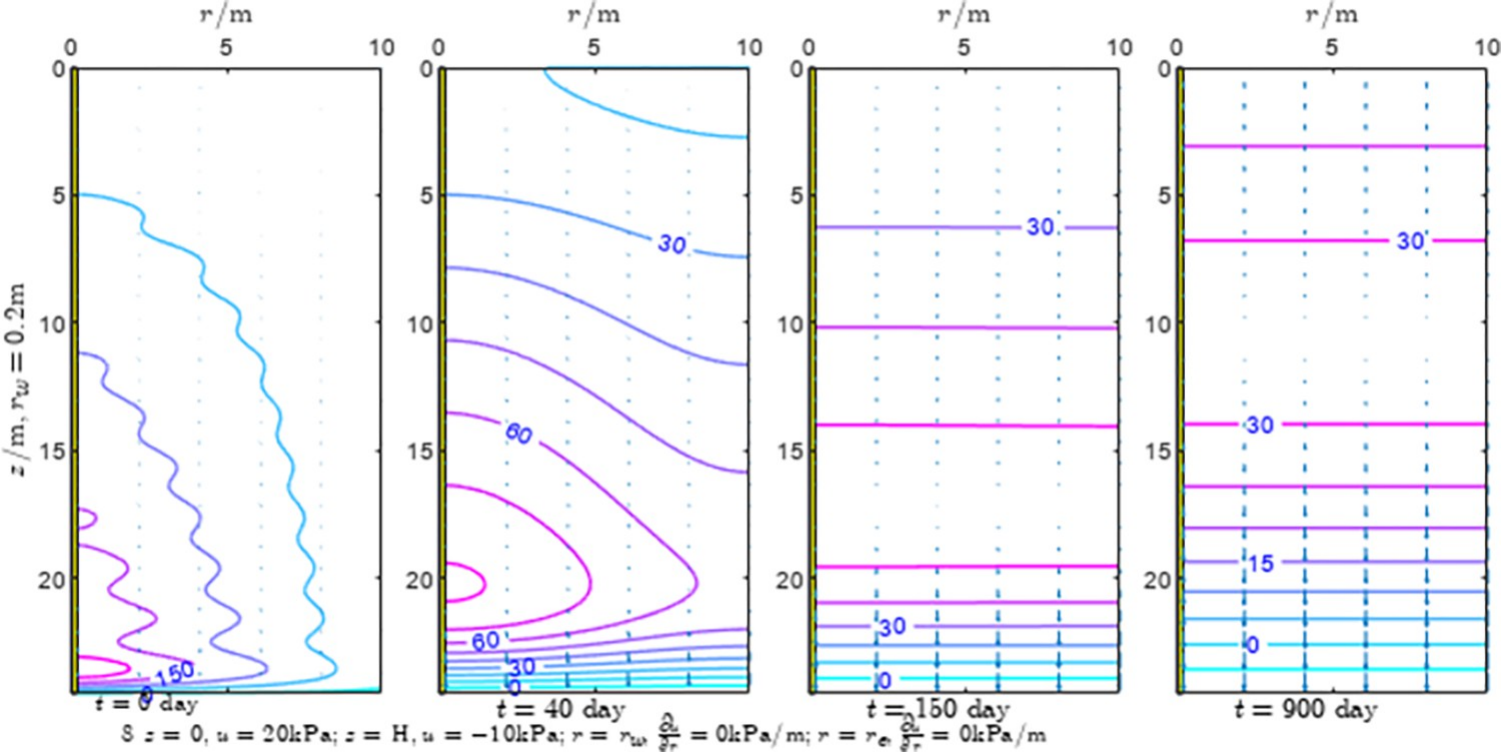
Spatial and temporal variation of consolidation seepage when the well radius is 0.2m.

**Fig 12:**
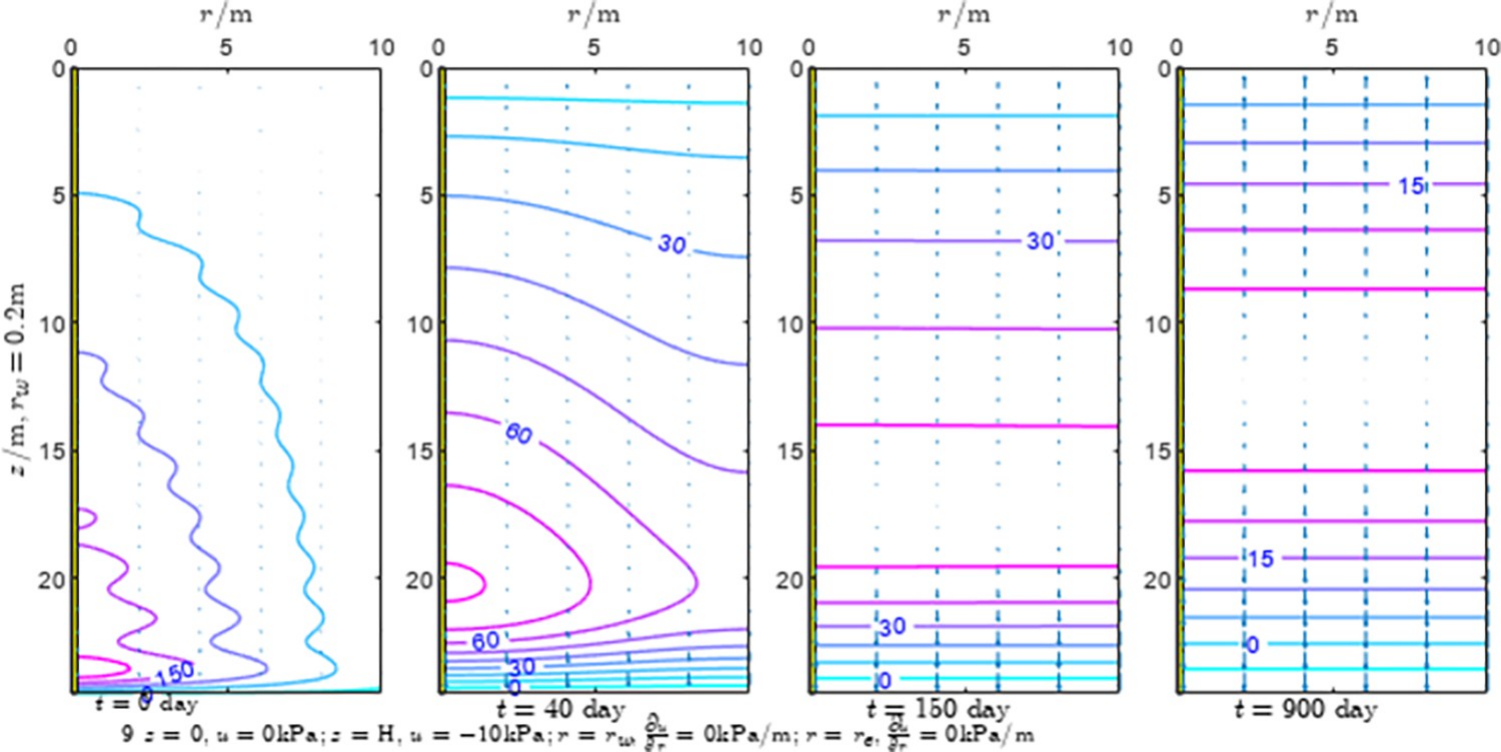
Spatial and temporal variation of consolidation seepage when the well radius is 0.2m.

**Fig 13:**
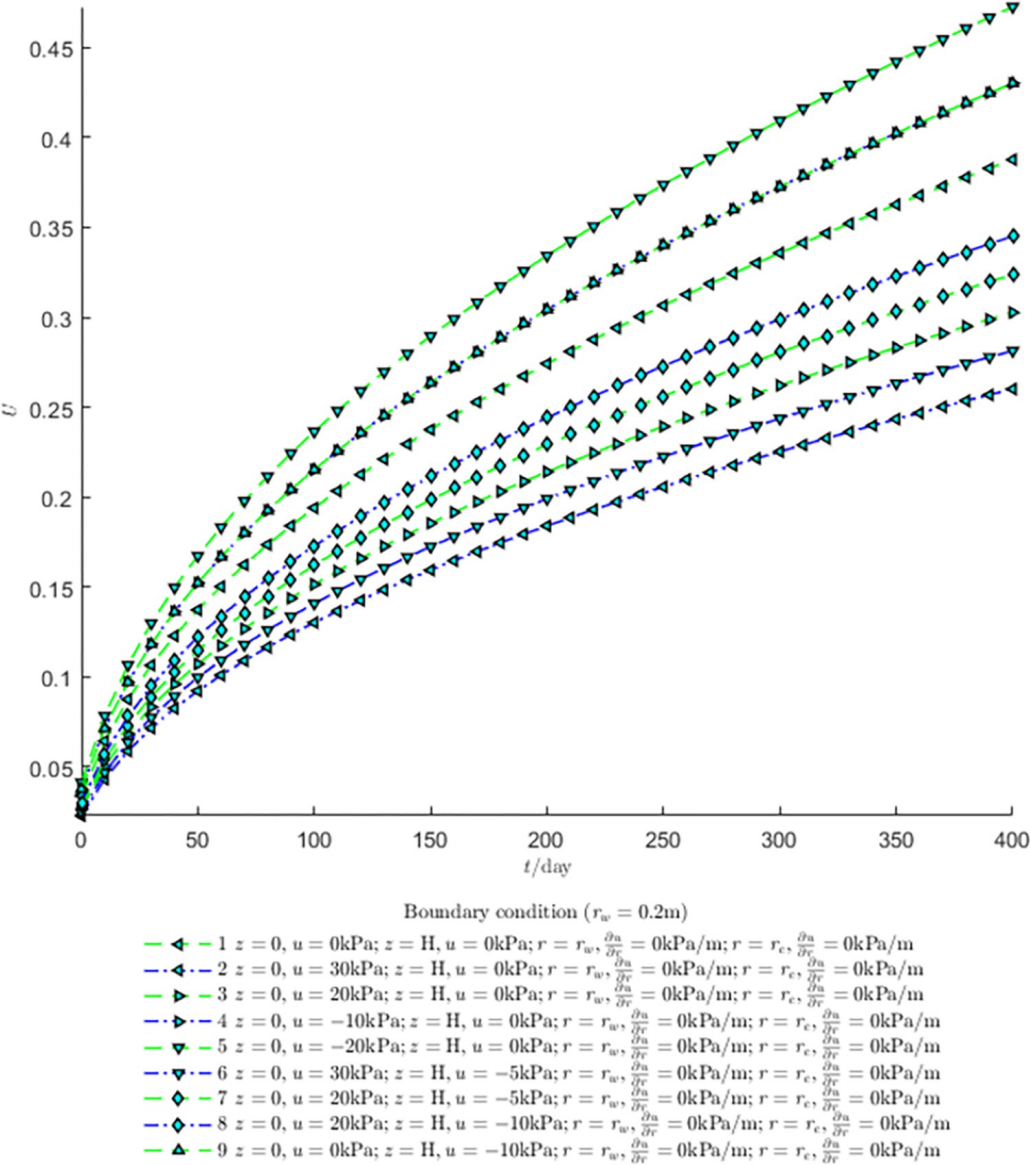
Effect of boundary on consolidation degree when the well radius is 0.2m.

According to Eqs (14–16), spatial and temporal variation of consolidation seepage and average degree when the well radius is 0.2m are calculated with solutions in enclosure space based on non-zero-constant values boundary. Then, the contour of spatial and temporal variation of the seepage are drawn based on the calculated data, as shown in Figs 4–12. The consolidation results are shown in Fig 13 and the calculation and contour results are analyzed as follows:

At *t* = 0, the contours can converge to the distribution law described by the initial function expressed as Eq (16), verifying the correctness of the solutions deduced in this paper.

For the three-dimensional case of ${\frac{\partial u}{\partial r}|}_{r=r_{w}}=0,{\frac{\partial u}{\partial r}|}_{r=r_{e}}=0$, the radial velocity must be 0 at *r* = *r*_*w*_ and *r* = *r*_*e*_, and comparing from the Figs 4–12, the solutions in this paper can conform to the requirement of 0 radial seepage velocity at the boundaries.

Average degrees of consolidation curves are shown in Fig 13. Boundary values affects the final values of dissipation degrees, and the negative water pressure increases the speed of consolidation.

Analyzing Figs 4–12, near the boundary of re = 10m, the initial water pressure is close to zero, according with the setting of the initial condition function expressed in Eq (16). At t >150 day, the velocity vector component tends to 0 along the *r* direction in the whole calculation area, which also conforms to the boundary condition ${\frac{\partial u}{\partial r}|}_{r=r_{w}}=0,{\frac{\partial u}{\partial r}|}_{r=r_{e}}=0$.

Analyzing the consolidated flow diagram, the arrow indicates the direction of the flow velocity vector, and the length of the arrowline indicates the size of the flow velocity. At the same time, the variation law of the isobar of excess pore pressure with time is also illustrated. The velocity direction is always the normal direction of the flow line at that time, verifying the correctness of the solutions deduced in this paper.

The theoretical solution using two-dimensional infinite series can simulate the instantaneous discontinuity between the boundary condition values and the initial condition values at the boundary for pore water pressure values. However, in practice, a large number of series terms need to be calculated to achieve this. In cases where computational resources are limited, significant computational errors may exist, as shown in the contour map for Day 0 in Figs 4–12.

As shown in Fig 13:

(1) The water pressure value at the recharge and the drainage boundaries determine the consolidation rate at a certain time. The average consolidation degree of soil layer in 400 days under condition 2 is half of that under condition 5. Because the site is located in a closed environment, the consolidation speed is slow overall.

(2) The average consolidation curves of working conditions 9 and 4 is basically overlapped, indicating that under the initial conditions in this paper, the boundary pressure values on the top and bottom of the same soil layer have basically the same effect.

(3) When the boundary water pressure value is positive, the average consolidation degree of soil layer at the same time decreases. When the boundary pressure value is negative, the consolidation speed drops and the average consolidation degree of soil layer at the same time increases.

(4) The calculated changes of soil consolidation degree with time and boundary conditions conform to the recharge or drainage hypothesis, which verifies the correctness of the theoretical solution.

## 4. Degenerate to unidirectional consolidation solution

The solution in this paper is a general solution obtained under the condition that the initial condition can be any function. When the initial condition is constant and the soil between piles is assumed to be isotropic, the solution can be reduced to the solution of the Terzaghi unidirectional consolidation problem. The reason of the long consolidation process between piles is explained under the conditions.

Eq (14) is a general solution, and its initial condition can be any function, and the top and bottom surfaces of the consolidated soil layer can be any constant. In particular, when *u*(*r*,*z*,0)=*u*_0_ is constant and *u*|_*z* = 0_ = 0,*u*|_*z* = *H*_ = 0,

$$\int_{r_{0}}^{r_{e}}\int_{0}^{H}u_{0}M_{i}\sin(\sqrt{\mu_{k}}z)rdrdz=0,$$

Therefore, from Eq (13) we know that *C*_*k*,*i*_ = 0 and,

$$C_{k,0}=\frac{\int_{r_{0}}^{r_{e}}\int_{0}^{H}u_{0}r\sin(\sqrt{\mu_{k}}z)drdz}{\int_{r_{0}}^{r_{e}}\int_{0}^{H}r\sin^{2}(\sqrt{\mu_{k}}z)drdz}=\frac{4u_{0}}{\pi (2k-1)}$$

By substituting *C*_*k*,*i*_,*C*_*k*,0_ into Eq (14) and assuming $n=\frac{C_{v}}{C_{h}}=1$, the Terzaghi unidirectional consolidation Eq (17) can be obtained.

$$u(r,z,t)=\sum_{k=1}^{\infty }\frac{4u_{0}}{\pi (2k-1)}\sin(\sqrt{\mu_{k}}z)e^{-\lambda_{k,0}C_{h}t}$$ (17)

From the above analysis, it can be seen that when the initial pore water pressure is uniformly distributed and *u*|_*z* = 0_ = 0,*u*|_*z* = *H*_ = 0, Eq (14) can be degraded to the solution of the unidirectional consolidation, which indirectly verifies the rationality of the derivation of the above solution.

## 5. Finite Element Methods (FEM) Simulation and verification

**Fig 14:**
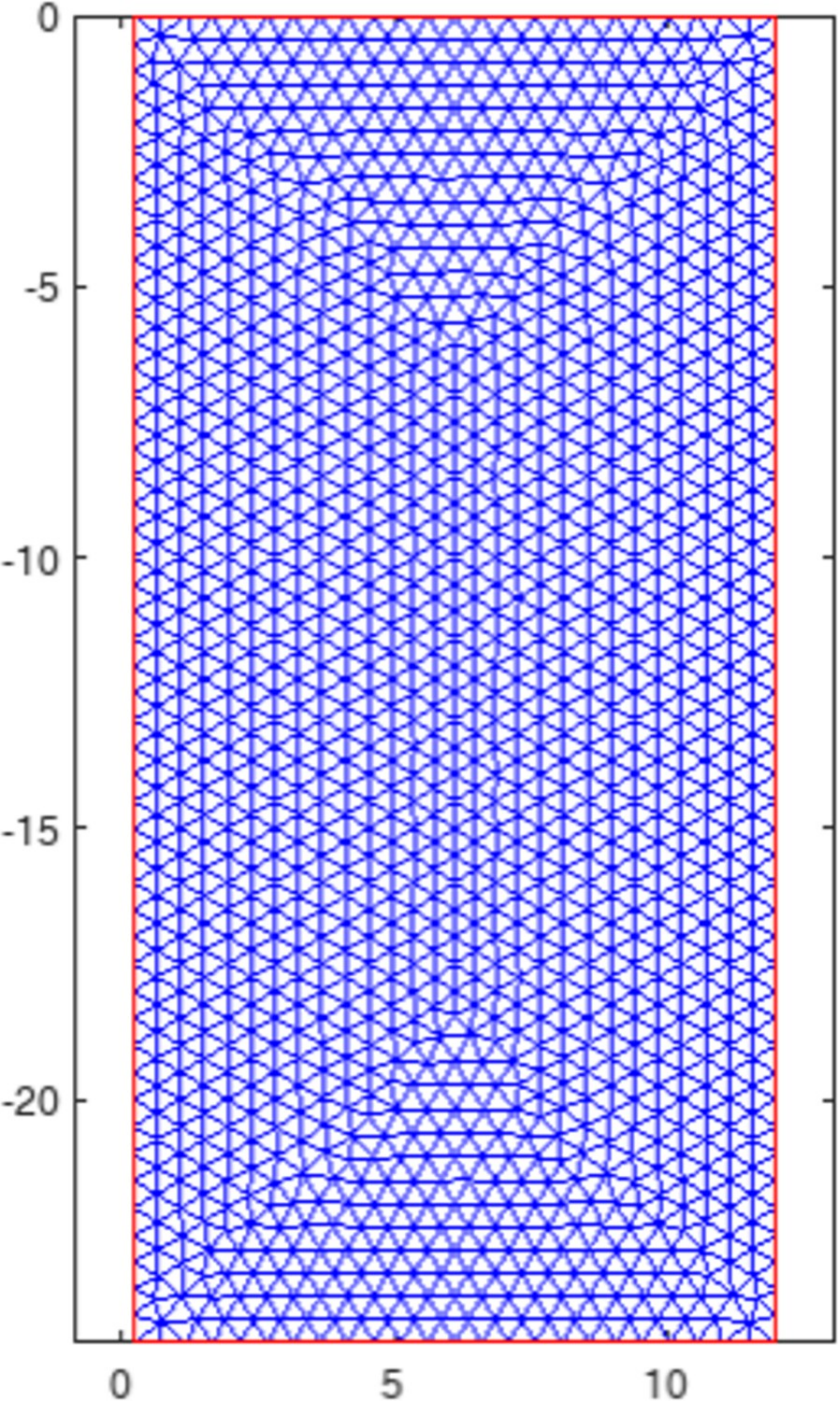
FEM model.

**Fig 15:**
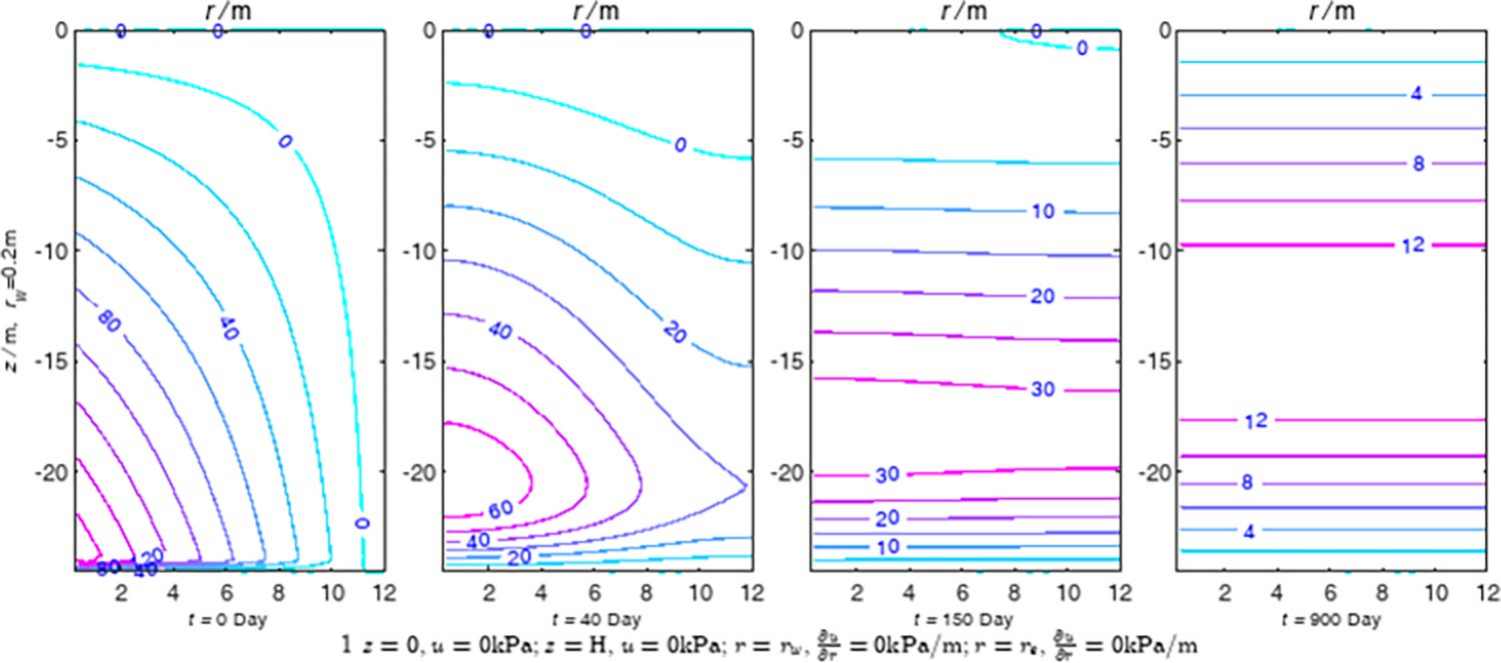
Results of FEM for constant values boundary condition 1.

**Fig 16:**
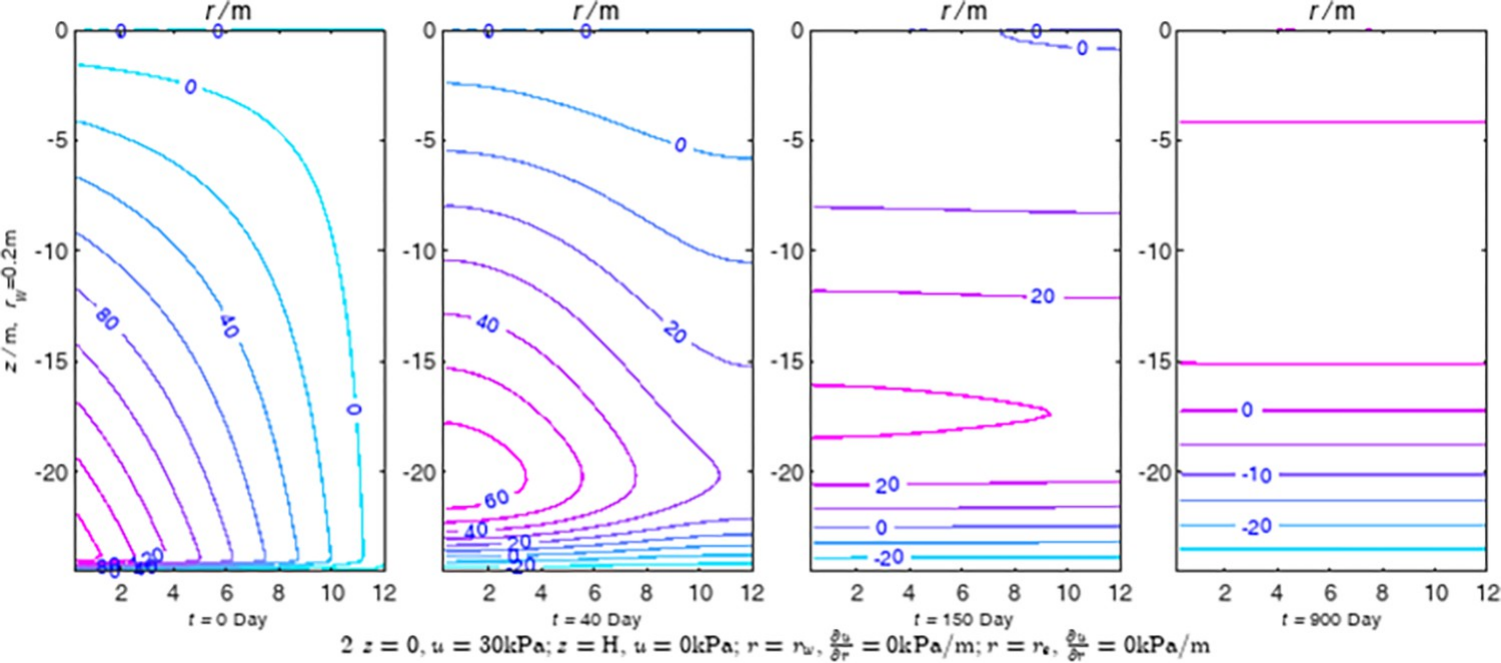
Results of FEM for constant values boundary condition 2.

**Fig 17:**
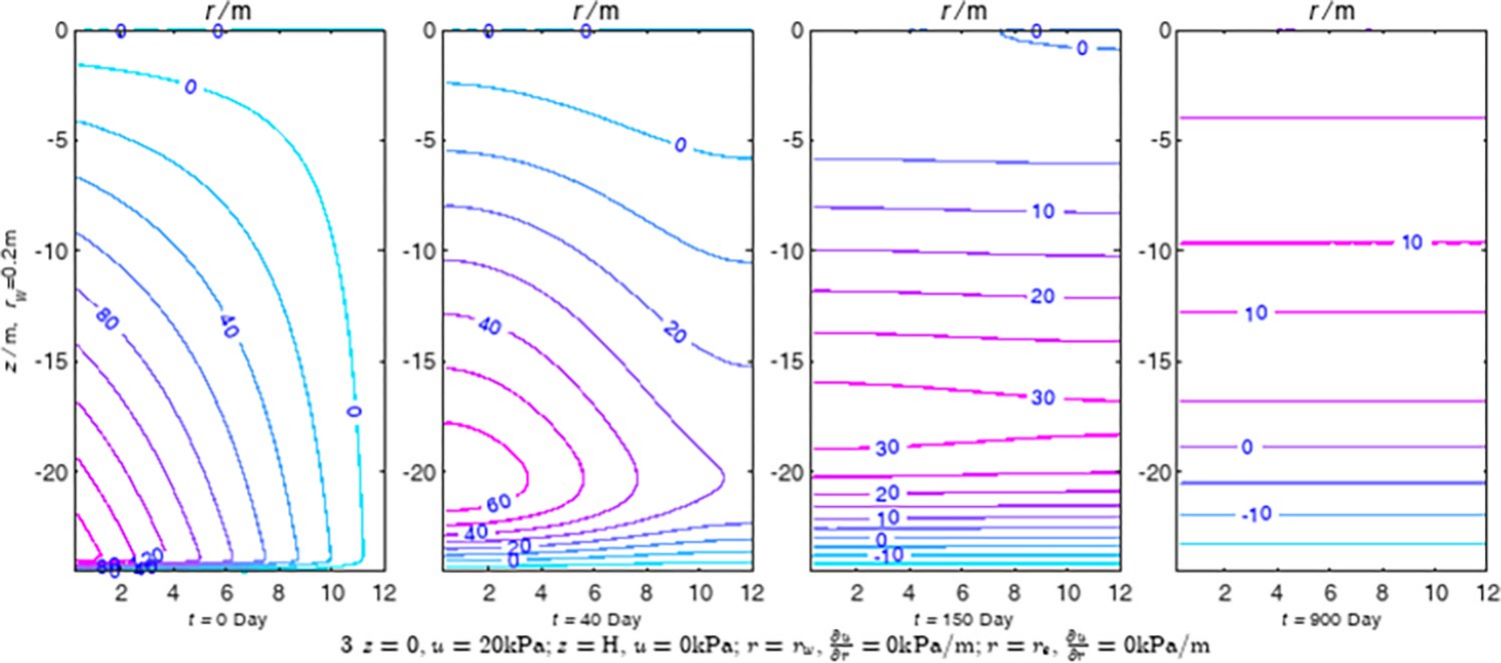
Results of FEM for constant values boundary condition 3.

**Fig 18:**
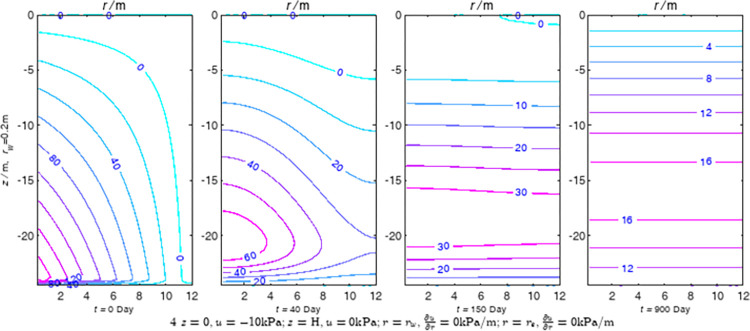
Results of FEM for constant values boundary condition 4.

**Fig 19:**
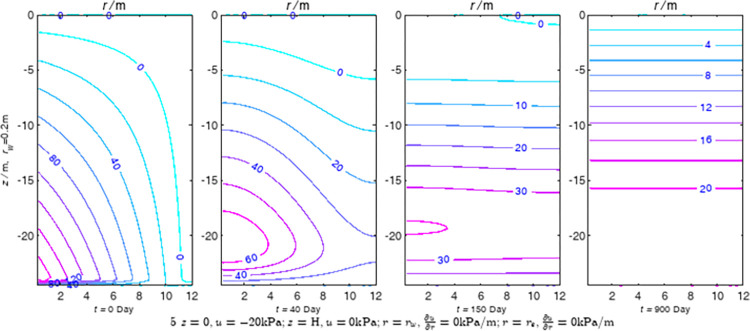
Results of FEM for constant values boundary condition 5.

**Fig 20:**
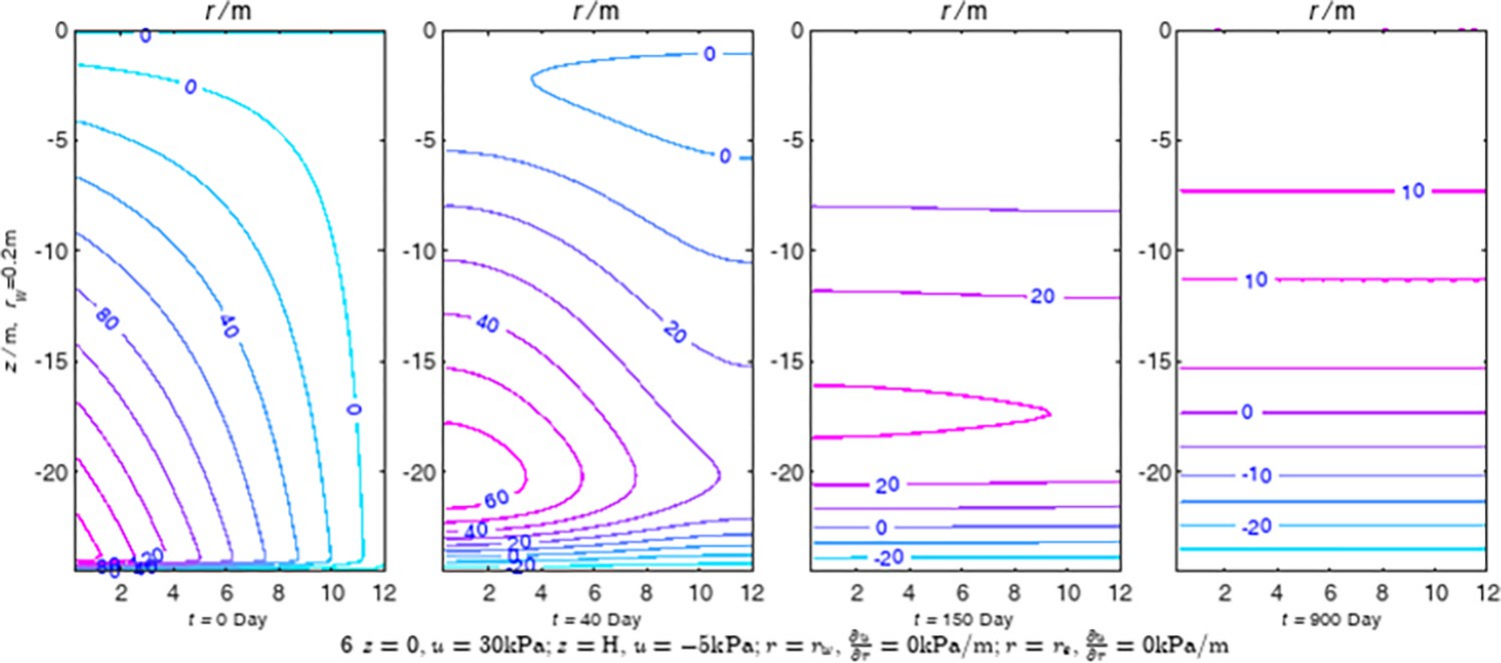
Results of FEM for constant values boundary condition 6.

**Fig 21:**
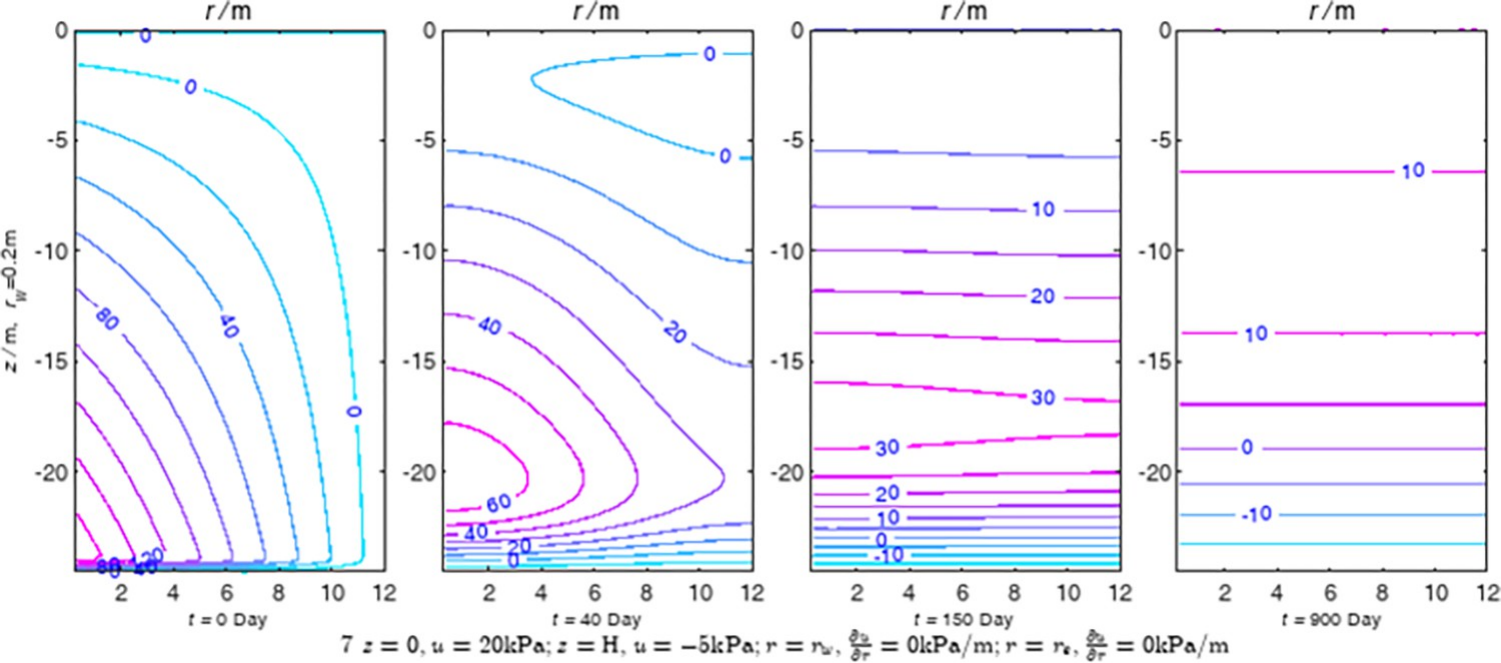
Results of FEM for constant values boundary condition 7.

**Fig 22:**
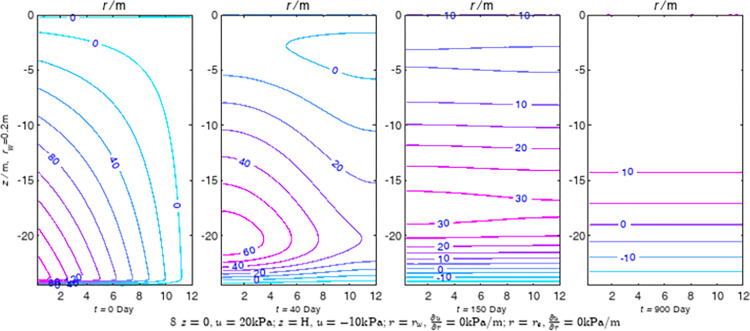
Results of FEM for constant values boundary condition 8.

**Fig 23:**
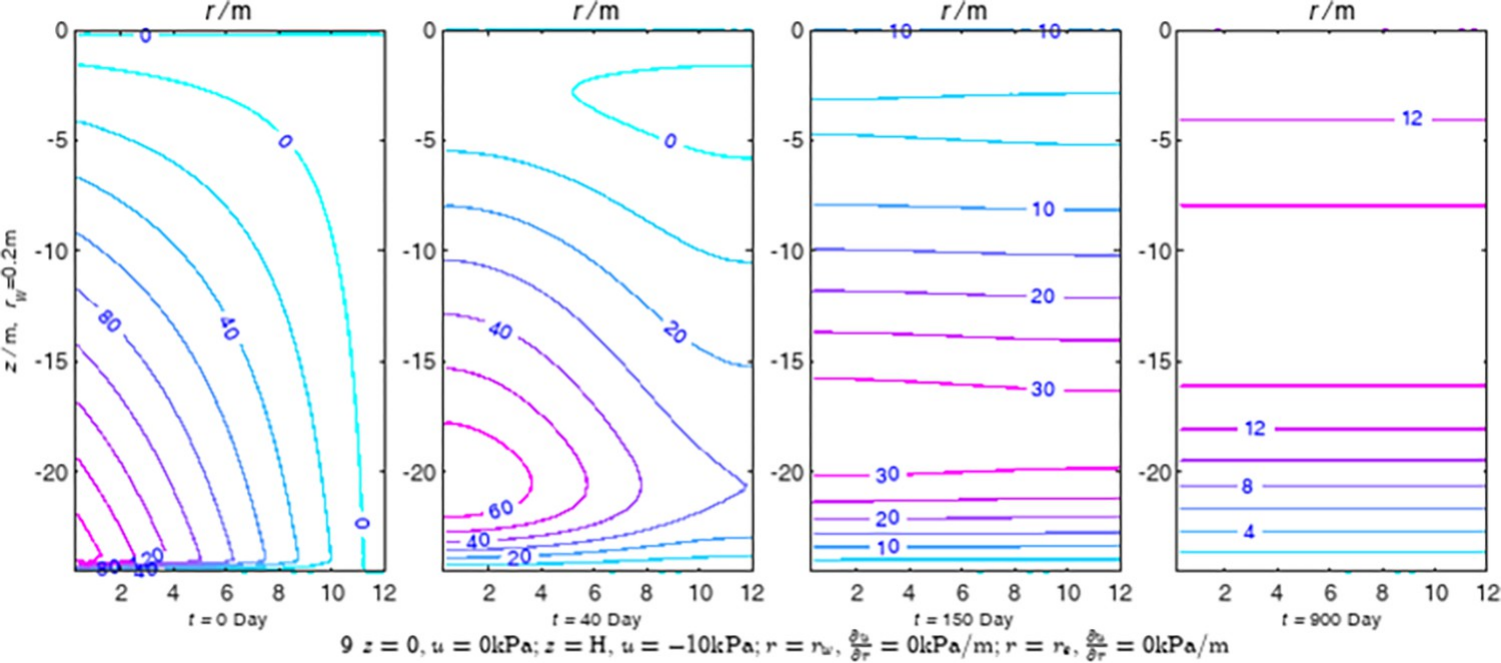
Results of FEM for constant values boundary condition 9.

Axisymmetric consolidation problems involve the dissipation of pore water pressure, a critical aspect in geotechnical engineering. Finite element simulation plays a crucial role in studying this phenomenon. By utilizing numerical methods, we can analyze the dissipation law of pore water pressure and its impact on soil behavior, providing insights into the mechanisms governing this process and enhancing our understanding of soil consolidation theoretical solutions deduced in this paper. In order to verify the correctness and convergence of the infinite series analytical solution derived in the article, a finite element model shown in Fig 14 is established based on the geometric and mechanical parameters determined by the above theory calculations. By strictly following the boundary conditions and initial conditions determined by the above theoretical calculations, results from finite element calculations can be obtained as shown in Figs 15–23.

For the axisymmetric consolidation and pore water pressure dissipation issues, the contour plots of the theoretical analytical results (Figs 4–12) and the FEM numerical simulation results (Figs 15–23) reflect similar patterns, indicating the correctness of the formula derivation in this paper. Although there are systematic deviations in the specific contour values, especially with larger discrepancies in the initial stages, analysis suggests that the deviations are primarily caused by the convergence rate of the two-dimensional infinite series solution.

## 6 Conclusions

Partial differential equation is established for consolidation and seepage solutions in enclosure space based on non-zero-constant values boundary, obtaining the theoretical solution for dissipation law of excess pore water pressure.

The spatio-temporal variation rules are analyzed to verify the correctness of the solutions obtained based on the percolation profile and velocity vector graph.

From the above analysis, it can be seen that when the initial pore water pressure is uniformly distributed the general solution can be degraded to the solution of the unidirectional consolidation problem, which indirectly verifies the rationality of the derivation of the solution.

The three-dimensional series solution of the horizontal isotropic soil consolidation between pile groups caused by static pile compression in saturated soft soil under closed environment is derived, which can effectively calculate and predict the excess pore water pressure and the consolidation degree of the study area at any time in the soil between pile groups after pile formation.

The excess pore water pressure and the degree of consolidation can be further used to calculate the bearing capacity of piles in saturated soft soil. In particular, it is difficult to quantitatively determine the bearing capacity aging of pile group foundation by experiment, so the time effect of bearing capacity is converted by calculating the consolidation degree of pile group foundation, which provides an effective way to solve this problem.

The series solutions of this paper have the value of engineering application and the significance of further theoretical research.
